# VMP1: a multifaceted regulator of cellular homeostasis with implications in disease pathology

**DOI:** 10.3389/fcell.2024.1436420

**Published:** 2024-07-19

**Authors:** Jia Tong, Qianqian Wang, Ziyan Gao, Yang Liu, Chengbiao Lu

**Affiliations:** ^1^ The Third Affiliated Hospital of Xinxiang Medical University, Xinxiang, Henan, China; ^2^ Henan Key Laboratory of Biological Psychiatry (Xinxiang Medical University), The Second Affiliated Hospital of Xinxiang Medical University, Xinxiang, Henan, China; ^3^ Henan International Joint Laboratory for Non-Invasive Neural Modulation, Department of Physiology and Pathology, School of Basic Medical Science, Xinxiang Medical University, Xinxiang, Henan, China; ^4^ Institute of Psychiatry and Neuroscience, Xinxiang Medical University, Xinxiang, Henan, China

**Keywords:** VMP1, autophagy, lipid scramblases, ER stress, coronavirus

## Abstract

Vacuole membrane protein 1 (VMP1) is an integral membrane protein that plays a pivotal role in cellular processes, particularly in the regulation of autophagy. Autophagy, a self-degradative mechanism, is essential for maintaining cellular homeostasis by degradation and recycling damaged organelles and proteins. VMP1 involved in the autophagic processes include the formation of autophagosomes and the subsequent fusion with lysosomes. Moreover, VMP1 modulates endoplasmic reticulum (ER) calcium levels, which is significant for various cellular functions, including protein folding and cellular signaling. Recent studies have also linked VMP1 to the cellular response against viral infections and lipid droplet (LD). Dysregulation of VMP1 has been observed in several pathological conditions, including neurodegenerative diseases such as Parkinson’s disease (PD), pancreatitis, hepatitis, and tumorogenesis, underscoring its potential as a therapeutic target. This review aims to provide an overview of VMP1’s multifaceted roles and its implications in disease pathology.

## 1 Introduction

Autophagy, a conserved cellular pathway, is fundamental to the maintenance of cellular homeostasis through the degradation and recycling of intracellular components (Klionsky, 2005; Mizushima and Komatsu, 2011; Lin et al., 2024). It plays a critical role in various physiological processes and has been implicated in the pathology of a wide spectrum of diseases, including neurodegenerative disorders (Liénard et al., 2024; Panwar et al., 2024) and cancer (Mathur et al., 2024; Rajendran et al., 2024; Yang et al., 2024). VMP1, a transmembrane protein that orchestrates key steps in the autophagic process, from the nucleation of autophagosomes to their fusion with lysosomes for cargo degradation (Ropolo et al., 2007; Mizushima and Komatsu, 2011; Hamasaki et al., 2013; Parzych and Klionsky, 2014; Wang et al., 2020b; Wang et al., 2021b; Dong et al., 2021; Jiang et al., 2022).

VMP1’s functions extend beyond autophagy, with emerging evidence highlighting its significance in modulating ER calcium levels, which are paramount for protein folding, cellular signaling, and responses to viral infections (Wang et al., 2021b; Jiang et al., 2022).

This review aims to dissect the intricate roles of VMP1 in cellular physiology and disease pathology by exploring VMP1’s involvement in autophagosome formation, regulation of ER calcium homeostasis, and the lipid metabolism. Furthermore, the interplay between VMP1 and microRNAs (miRNAs), which are known to modulate its expression and contribute to cancer progression, will be discussed. By elucidating the functions of VMP1 and its regulatory mechanisms, we hope to provide a comprehensive overview of this integral protein and its relevance to human health and disease.

### 1.1 VMP1 contributes to autophagosome formation

Autophagosome formation involves the nucleation of a cup-shaped membrane, known as the isolation membrane (IM), originally termed as phagophore or autophagosome precursor, and its further expansion and closure coordinated with ATG (autophagy-related gene) proteins (Lamb et al., 2013) to form the autophagosome (Polson et al., 2010; Feng et al., 2014).

**FIGURE 1:**
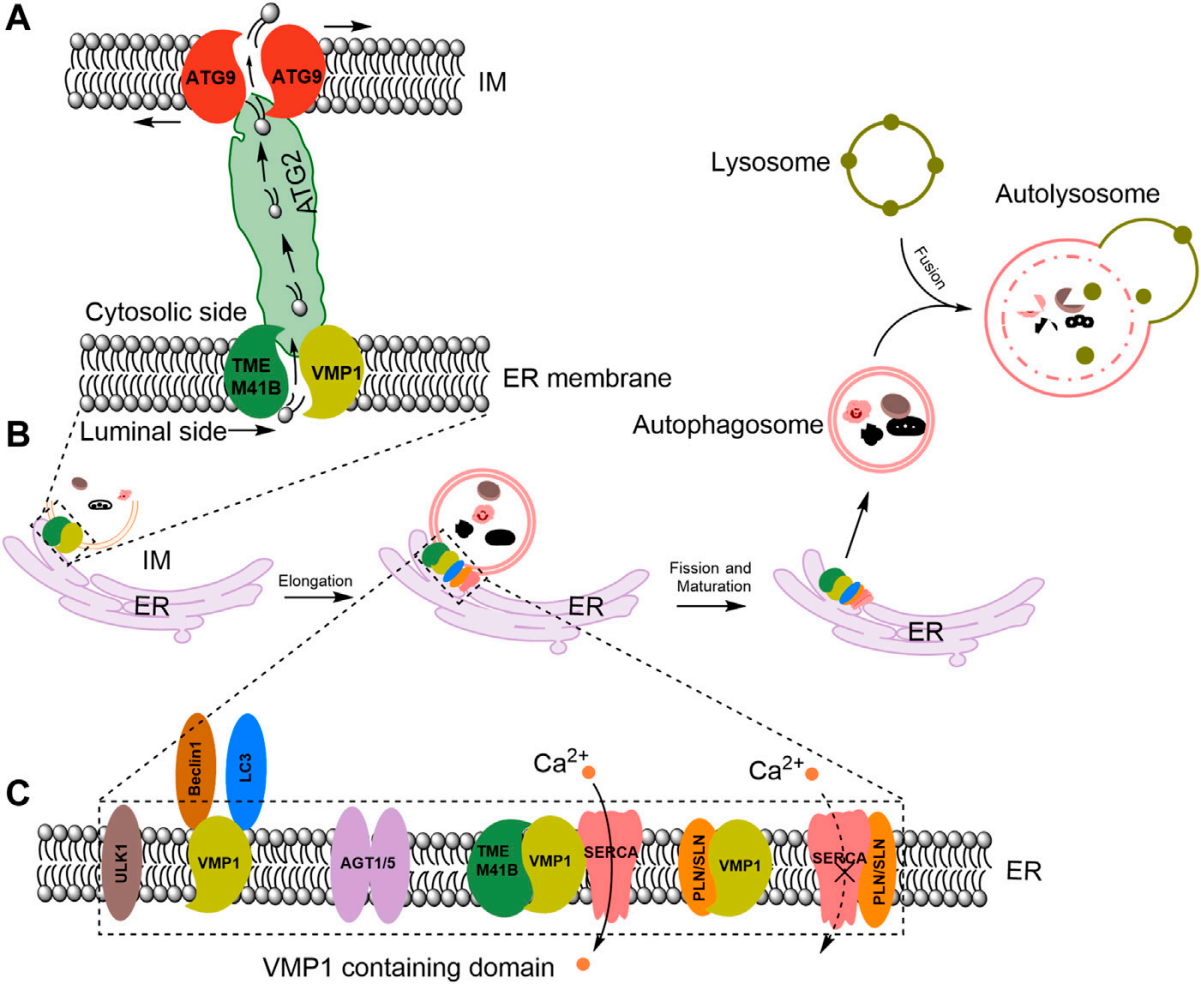
VMP1 mediates ER-IM contact and acts as a lipid scramblase in autophagosome formation and maturation. **(A)** Proposed model for VMP1 and TMEM41B in facilitating lipid transport from the ER to autophagosomes. ATG, autophagy-related. **(B)** VMP1 mediates autophagosomes maturation. **(C)** Molecular regulators recruited to ER-IM contacts during autophagosome formation. PLN/SLN: phospholamban/sarcolipin; ULK1: Unc-51 like autophagy activating kinase 1; LC3: microtubule-associated protein one light-chain 3. ATG1/5: autophagy-related proteins one or 5. TMEM41B: transmembrane protein 41B.

VMP1, a transmembrane protein featuring six hydrophobic regions, acts as an important modulator in autophagosome formation. As shown in Figure 1A, VMP1 interacts with another autophagy factor, ER transmembrane protein 41B (TMEM41B) to regulate IM and autophagosome formation (Morita et al., 2018; Ghanbarpour et al., 2021; Reinisch et al., 2021).

*TMEM41B* is identified as a novel *ATG* gene (Morita et al., 2018). Deletion of TMEM41B blocks autophagosome formation at an early step (Moretti et al., 2018), causing accumulation of ATG proteins and lipid droplets (LDs) accumulation (Carlini et al., 2024).

Under physiological conditions, VMP1 and TMEM41B form a 1:1 complex (Morita et al., 2018) and share overlapping functions in the autophagy process. They act locally and function as components of a sophisticated lipid transportation mechanism at the contact sites between the ER and autophagosomes, facilitating the transfer of lipids from the ER to the growing autophagosome membrane (Ghanbarpour et al., 2021; Reinisch et al., 2021) (Figure 1A).

ATG2 function as bridge that allows for bulk lipid flow between ER and nascent autophagosomes (Valverde et al., 2019) (Figure 1A). And ATG9 facilitates the integration of incoming lipids into the autophagosome membrane and re-equilibrates the cytosolic and luminal leaflets to allow for membrane growth (Matoba et al., 2020; Ghanbarpour et al., 2021) (Figure 1A). In mammalian cells, VMP1-containing domains of the ER have been shown to recruit the homolog of ATG1, Unc-51 like autophagy activating kinase one complex (ULK1) and ATG5 complexes (Itakura and Mizushima, 2010). VMP1 interacts with Beclin1, a mammalian autophagy initiator, and recruits the microtubule-associated protein one light-chain 3 (LC3), a marker of the autophagosomes (Ropolo et al., 2007) to induce the formation of autophagosome (Figures 1B, C).

VMP1 enhances sarco/ER Ca2+-ATPases (SERCA) activity to regulate ER-IM contacts, preventing the inhibitory SERCA/phospholamban/sarcolipin complex formation and sustaining a low calcium environment at the contact site (Figure 1C). This low calcium environment is essential for autophagosome formation and maturation (Vandecaetsbeek et al., 2011). Autophagosomes fuse with lysosomes to form autolysosomes (He and Klionsky, 2009; Chen and Klionsky, 2011) (Figure 1B). Thapsigargin (TG), an inhibitor of the ER SERCA calcium pump (Thastrup et al., 1989; Lewis, 2011), prevents the final closure and detachment of autophagosomes from the ER (Engedal et al., 2013), leading to accumulation of abnormal autophagosomes at the ER (Ganley et al., 2011). However, the precise mechanism by which Ca^2+^ influence the formation of autophagosomes still requires further investigation.

### 1.2 VMP1 mediates calcium homeostasis in response to ER stress

While VMP1’s contribution to the formation of autophagosomes is pivotal, it also plays a critical role in maintaining the balance of Ca^2+^ within the cell. ER is central to cellular calcium homeostasis. Disruptions in ER calcium balance initiate stress responses (Groenendyk et al., 2021; Proulx et al., 2021; Groenendyk and Michalak, 2023; Nagar et al., 2023). The SERCA pumps calcium from cytosol ([Ca^2+^] ∼100 nM) into ER ([Ca^2+^] ∼100 μM–1 mM) against calcium concentration gradient (Wuytack et al., 2002). Whereas VMP1 mediates the efflux of calcium from ER lumen into cytoplasm, thereby maintaining the steady state of calcium homeostasis of ER. VMP1 deficiency leads to ER calcium overload (Zhao et al., 2017), whereas overexpression of VMP1 depletes ER calcium, with aspartic acid 272 identified as a key residue for VMP1’s ER calcium releasing activity (Liu et al., 2023). VMP1 deletion induces ER calcium overload, which also leads to mitochondria calcium overload (Zack et al., 2024), because mitochondria take up Ca^2+^ from the ER via the mitochondrial calcium uniporter at the mitochondrion-ER contact sites (Patergnani et al., 2011). Mitochondrial Ca^2+^ overload is known to trigger cell death (Giorgi et al., 2012). Although recent studies suggest that VMP1 may function as a novel calcium-permeable protein (Liu et al., 2023), definitive evidence necessitates additional research endeavors, such as the reconstitution of VMP1 into recombinant liposomes for patch-clamp analysis.

In addition, VMP1 acts as a pivotal regulator of ER stress sensitivity, its deficiency induces ER calcium overload and triggers ER stress, which initiates the unfolded protein response (UPR) (Zhao et al., 2017). The UPR is initiated and regulated by three ER sensors: inositol-requiring enzyme 1 (IRE1), protein kinase R-like ER kinase (PERK), and activating transcription factor 6 (ATF6). Specifically, deletion of VMP1 activates PERK under basal conditions but suppresses IRE1a and ATF6 pathways in response to ER stress (Li et al., 2023). This nuanced regulation by VMP1 commits limited activation of UPR yet refrains from UPR hyperactivation, which is crucial for protecting cells from chronic ER stress.

### 1.3 VMP1 plays a role in lipid metabolism

ER, a central hub for lipid synthesis and calcium homeostasis, is also the site where VMP1 exerts its influence on lipid metabolism. VMP1 also collaborates with TMEM41B to form a functional complex integral to the ER’s role in lipid homeostasis (Morita et al., 2019; Reinisch et al., 2021). Recent studies also suggest that both VMP1 and TMEM41B are lipid scramblases (Ghanbarpour et al., 2021; Li et al., 2021; Reinisch et al., 2021; Hama et al., 2022), capable of regulating the asymmetric distribution of lipids and the curvature of membranes (Tang et al., 2022; Wang and Kinoshita, 2023). Depletion of either scramblase leads to a mis-sorting of both phosphatidylserine and cholesterol (Li et al., 2021).

Besides, both TMEM41B and VMP1 are crucial for LDs formation and apolipoprotein production on the ER. Depletion of either of the two genetically leads to the buildup of enlarged LDs (Tábara and Escalante, 2016; Moretti et al., 2018), as well as the formation of apolipoproteins that are either incomplete or absent, resulting in a subsequent impairment of lipid secretion that relies on these apolipoproteins (Morishita et al., 2019; Huang et al., 2021). In the context of LD formation, studies have demonstrated that VMP1 is located in close proximity to the sites where LDs are budding (Tábara and Escalante, 2016; Zhao et al., 2017). The precise mechanism of VMP1 localization to these areas remains unclear.

### 1.4 Post-translational regulation of VMP1

Ubiquitination is a critical post-translational modification involved in multiple cellular pathways, which takes place through the sequential action of three enzymes: the ubiquitin-activating enzyme (E1), the ubiquitin-conjugating enzyme (E2), and the ubiquitin protein ligase (E3) (Hershko and Ciechanover, 1998; Schulman, 2011). As others have reviewed, most autophagy-related proteins are regulated by ubiquitination (Chen et al., 2019).

VMP1 can be modified by polyubiquitination not monoubiquitination, and the cullin-RING E3 ubiquitin ligase CRL4/Cdt2 is involved in this ubiquitination process, resulting in the degradation of VMP1 and a decrease in VMP1-mediated autophagy (Renna et al., 2023). VMP1 is ubiquitinated early in autophagosome biogenesis and remains ubiquitinated as part of the autophagosome membrane throughout the autophagic flux until autolysosome formation (Renna et al., 2023). On the other hand, limited reports have indicated that VMP1 interacts with ubiquitin specific peptidase 20 (USP20) (Zhang et al., 2024) and USP9x (Grasso et al., 2011) at the ER membrane, which are responsible for its deubiquitination. However, the specific mechanisms by which the ubiquitination-deubiquitination dynamic of VMP1 regulates autophagy and protein degradation pathways remain to be further clarified.

Besides undergoing ubiquitination, recent studies have also reported that VMP1 can be palmitoylated at cysteine residues 263 and 278 by ZDHHC3 (Qu et al., 2024). This modification is crucial for the subcellular localization and functional regulation of VMP1. Mutations at the palmitoylation modification sites result in diminished plasma membrane localization of VMP1. And also, palmitoylation of VMP1 enhanced the secretion of small extracellular vesicles, influencing the self-renewal capacity of small cell lung cancer cells and their apoptotic and proliferative behaviors. Disruption of VMP1’s palmitoylation sites impairs the biogenesis of multivesicular bodies and the formation of intraluminal vesicles (Qu et al., 2024).

### 1.5 VMP1 and diseases

Given the pivotal roles that VMP1 plays in cellular processes, it is not surprising that alterations in VMP1 function is implicated in various pathological states. Understanding the precise mechanisms by which VMP1 dysfunction leads to disease will be critical for identifying novel therapeutic targets and strategies for intervention.

#### 1.5.1 Neurodegenerative diseases

Autophagosome-lysosome pathway (Ravikumar et al., 2002; Martinez-Vicente and Cuervo, 2007; Pan et al., 2008; Koh et al., 2019) and ubiquitin-proteasome system (Sawada et al., 2004; Olanow and McNaught, 2006) are the most important machineries responsible for protein degradation in Parkinson’s disease (PD) (Shen et al., 2013; Singh et al., 2022) and amyotrophic lateral sclerosis (Şentürk et al., 2019). Basal autophagy is crucial for the clearance of diffuse cytoplasmic proteins, thereby preventing protein aggregation and preserving neuronal integrity. Disruption of this process, as evidenced by the work of Hara et al. (2006), can lead to impaired motor function and the accumulation of cytoplasmic inclusions in neurons. Specifically, VMP1 has been identified as a critical factor in PD pathogenesis.

Mice with targeted VMP1 deficiency in midbrain dopaminergic neurons progress to display escalated motor deficit alongside neuronal loss, synaptic dysregulation, and of α-synuclein aggregation (Wang et al., 2021b). Additionally, VMP1 deficiency is linked to cellular disturbances, including the formation of large vacuole-like structures, impaired mitochondrial function, and the accumulation of ubiquitin-positive aggregates(Calvo-Garrido and Escalante, 2010; Wang et al., 2021b). Clinical studies have correlated lower VMP1 levels in peripheral blood mononuclear cells of PD patients with disease severity, suggesting a potential diagnostic or therapeutic target. Notably, the expression levels of VMP1 decrease in correlation with the progression of PD; however, this reduction can be mitigated through the administration of agonists targeting dopamine receptors (Al-Nusaif et al., 2022).

#### 1.5.2 Tumor occurrence

Emerging evidence also suggests a tight link between autophagy and the progression of tumorigenesis (Rakesh et al., 2022). It serves a dual function in oncology: autophagy can both hinder malignant transformation and, paradoxically, facilitate cancer progression (Folkerts et al., 2019; Ding et al., 2024). VMP1, has been implicated in the regulation of autophagy and is associated with multiple cancers. The initial studies indicate that VMP1 overexpression can lead to the formation of intracellular vacuoles and subsequent cell death (Dusetti et al., 2002).

Building on these early findings, VMP1’s expression patterns and its functional implications vary significantly across different cancer types, adding another layer of complexity to our understanding of its role in cancer biology. In pancreatic cancer, VMP1 is upregulated under stress conditions and is associated with Kirsten rat sarcoma viral (KRAS) oncogene activation, contributing to tumor initiation and chemoresistance (Lo Ré et al., 2012; Gilabert et al., 2013; Loncle et al., 2016; Ropolo et al., 2020; Bai et al., 2021). This trend is mirrored in glioma, where increased VMP1 expression is linked to malignancy and poor prognosis, and it promotes cell proliferation as well as resistance to both chemotherapy and radiotherapy (Lin et al., 2021). In contrast, in hepatocellular carcinoma, VMP1 exerts a protective role by decreasing tumor spread and improving patient prognosis (Ying et al., 2011; Guo et al., 2012). Amirfallah et al. (2019) observed elevated VMP1 expression in breast tumors, and VMP1’s potential fusion with the ribosomal protein S6 kinase- 70 kDa polypeptide 1, also known as RPS6KB1 gene, suggesting a role for VMP1 in the development of malignant tumors (Renna et al., 2024). However, in breast (Amirfallah et al., 2019; Park et al., 2023), colorectal (Qian et al., 2014; Guo et al., 2015; Wang et al., 2020a), gastric (Wang et al., 2021a; Wu et al., 2023), esophageal, and ovarian cancers (Liu et al., 2014; Zheng et al., 2016), the relationship between VMP1 expression and malignancy is less clear, with conflicting reports that underscore the necessity for further investigation into the genetic context of each tumor type (Renna et al., 2024).

Further complicating the role of VMP1 in cancer is its interaction with multiple miRNAs. MiRNAs are pivotal regulators of gene expression and are intimately linked to the development and progression of cancer (Volinia et al., 2006). MiR-21, miR-210, and miR-124 are three miRNAs that have been associated with the expression of the VMP1 gene, which is itself implicated in cancer pathophysiology. MiR-21 is frequently upregulated in cancer and is located near the 3′-untranslated region of the VMP1 gene. It is transcribed from within the VMP1 gene and its elevated levels are associated with increased cancer cell growth and reduced apoptosis (Löffler et al., 2007; Ozsolak et al., 2008). MiR-21’s overexpression can induce a malignant phenotype, while its deletion can reduce tumor formation, highlighting its oncogenic role. MiR-210 targets VMP1, leading to its downregulation (Ying et al., 2011; Liu et al., 2014; Cui et al., 2015; Yang et al., 2021), which is associated with a pro-tumorigenic effect across various cancers, including lung adenocarcinoma (Cui et al., 2015), ovarian cancer (Liu et al., 2014) and hepatocellular carcinoma (Ying et al., 2011). MiR-210 is induced by factors such as hypoxia and von Hippel–Lindau tumor inactivation, and its upregulation correlates with poor prognosis and increased metastasis. MiR-124 displays context-dependent roles in cancer (Liang et al., 2017). It is downregulated in colorectal cancer and its overexpression can inhibit cancer cell migration and invasion (Guo et al., 2020). In contrast, in acute lymphoblastic leukemia, miR-124 upregulation contributes to drug resistance (Liang et al., 2017). VMP1 is also reported to be regulated by miR-124a, which impacts muscle cell behavior in goats, suggesting a broader role for miR-124 in cellular processes beyond cancer (Liu et al., 2022).

The post-translational modification of VMP1, specifically its ubiquitination, has emerged as another layer of regulation that impacts autophagy in tumor cells (Renna et al., 2023). The Cullin-ring ubiquitin ligase 4—DDB1-CUL4 associated factors (CRL4-DCAF2) ubiquitin ligase complex, known to interact with VMP1, plays a crucial role in this process. The modulation of CRL4 activity, either through inhibition or the overexpression of its component DCAF2, can significantly alter the ubiquitination status of VMP1. This finding suggests that the ubiquitin-proteasome system may offer another avenue for therapeutic intervention in cancers where VMP1 plays a significant role.

#### 1.5.3 Viral infection

VMP1, identified as a host factor for viruses, has emerged as a topic of significant research interest in recent years. VMP1 and TMEM41B were reported to be critical in corona- and flavivirus replication. TMEM41B is an essential host factor facilitating infection by various coronaviruses, including SARS-CoV-2, HCoV-OC43, HCoV-NL63, and HCoV-229E, as well as several flaviviruses that are of significant public health concern (Schneider et al., 2021; Yousefi et al., 2022). Genome-wide screens revealed the autophagy proteins VMP1 and TMEM41B as important host factors for SARS-CoV-2 infection (Schneider et al., 2021). Poirier’s studies have further broadened our understanding of the phylogeny of host factors. They found that multiple sgRNAs targeting VMP1 were significantly enriched in the HCoV-229E screen, and VMP1 also validated in the orthogonal arrayed CRISPR experiments, indicating that VMP1 alone or in combination with other VTT-domain proteins acts as a host factor for human coronaviruses. This conclusion has also been further confirmed in subsequent studies. Using double membrane vesicle (DMV) induction via co-expression of SARS-CoV-2’s NSP3 and NSP4, Ji et al. (2023) found that VMP1 and TMEM41B are essential for DMV formation, with VMP1 mediating membrane closure and TMEM41B affecting NSP3/4 complex formation. Both VMP1 and TMEM41B are required for β-coronavirus infection and they function at distinct steps of DMV biogenesis (Ji et al., 2022). Interestingly, SARS-CoV-2 spike protein induces calcium oscillations that are critical for its infection of cells (Braga et al., 2021). Given the integral function of VMP1 in preserving ER calcium homeostasis and the ER’s pivotal role in the replication of viruses, it is plausible that VMP1 may exert regulatory control over viral infection via the modulation of ER calcium ions. Consequently, the modulation of VMP1 could present a promising therapeutic strategy for conditions characterized by dysregulation of ER calcium.

Additionally, VMP1 and TMEM18 also play significant roles in flavivirus infection. Where upon flavivirus infection and translation of the viral polyprotein, TMEM41B is recruited to sites on the ER membrane together with NS4A and NS4B where replication complexes are forming, and interacts with the VMP1 protein to form DMV. This interaction, facilitated by VMP1’s ability to alter membrane curvature, creates a protective environment for the virus’s replication (Hoffmann et al., 2021; Yousefi et al., 2022). Thus, TMEM41B has been nominated as a broad-spectrum RNA virus liability (Hoffmann et al., 2021) and potential high-priority target for future drug development efforts.

#### 1.5.4 Inflammation

Reduced VMP1 expression exacerbates inflammation (Grasso et al., 2011). VMP1 deficiency in monocytic cell lines leads to increased activation of the NLRP3 inflammasome, and caspase-1, as well as the release of proinflammatory molecules, such as IL-1β and galectin-3 (Zack et al., 2024). VMP1 was also involved in the cytoplasmic vacuolization of acinar cells during the early stage of acute pancreatitis (Dusetti et al., 2002; Vaccaro et al., 2003; Vaccaro et al., 2008). Knockout of VMP1 in mouse pancreatic acinar cells promotes inflammation and fibrosis, which is similar to the pathology of human chronic pancreatitis (Wang et al., 2022). Mechanistically, the absence of VMP1 leads to impaired autophagic flux and ER stress, as well as the activation of the NFE2L2/Nrf2 pathway (Wang et al., 2022). This pathway has a multifaceted influence on tissue injury and carcinogenesis (Wang et al., 2008; Dodson et al., 2019). Additionally, limited report shows that VMP1 also plays a significant role in mitophagy induced by acute pancreatitis (Vanasco et al., 2021).

Jiang et al. (2022) delineates VMP1 as a critical regulator of lipoprotein secretion and lipid homeostasis. VMP1 deficiency in mice recapitulates key features of non-alcoholic steatohepatitis, while VMP1 overexpression mitigates steatosis in non-alcoholic steatohepatitis, underscoring its essential role in the pathogenesis of non-alcoholic fatty liver disease. VMP1 gene konckout leads to the accumulation of lipoproteins within the hepatic and intestinal tissues of zebrafish, and a similar lipid accumulation is observed in the visceral endoderm and gut of VMP1-deficient mice (Morishita et al., 2019). These studies imply that VMP1 acts as a negative regulator of the inflammatory process, particularly during the pathogenesis of pancreatitis and non-alcoholic steatohepatitis.

Additionally, Herpes Simplex Virus Type 1 infection triggers microglial activation and neuroinflammation through the downregulation of VMP1 and upregulation of WHSC1L1, a shift that impairs mitophagy and amplifies neuroinflammation. This process is mediated by the epigenetic modification of VMP1, where WHSC1L1-induced H3K36me2 recruits DNMT3A, a DNA methylation enzyme, to repress VMP1 transcription, potentially involving methylation modifications of the VMP1 RNA (Yao et al., 2023).

## 2 Discussion

VMP1 is a pivotal regulator of cellular homeostasis, with its dysregulation implicated in a myriad of diseases, notably neurodegenerative disorders and cancer. This review has highlighted the multifaceted roles of VMP1 in regulating autophagy, maintaining calcium homeostasis, and responding to cellular stress and infection. VMP1’s role in neurodegenerative diseases is equally significant, with its potential as a diagnostic or therapeutic target in conditions like PD. The link between VMP1, ER stress, and the UPR pathways provides insights into how ER dysfunction can lead to protein aggregation and cellular demise.

VMP1 binds to Beclin1, which can shift the balance to induction of autophagy during oncogenesis (Molejon et al., 2013). The interplay between VMP1 and autophagy is particularly noteworthy, given autophagy’s dual role in oncogenesis. On one hand, autophagy serves as a survival mechanism for cancer cells under therapeutic stress, allowing them to evade apoptosis, and its inhibition can potentially enhance the effectiveness of anticancer treatments (Mahalingam et al., 2014; Rangwala et al., 2014; Vogl et al., 2014). On the other hand, autophagy can act as a promoter of cytotoxic cell death, and in such cases, inhibiting it might reduce the therapeutic benefits (Kim et al., 2015; Chen et al., 2016; Yu et al., 2017). Therefore, the decision to target autophagy in combination therapies hinges on its role as either a facilitator of survival or a driver of cell death.

VMP1 modulation by miRNAs contribute to the multifaceted aspects of cancer biology, influencing processes from cell proliferation to metastasis and patient outcomes. Their dysregulation is a common theme in cancer, underscoring their potential as therapeutic targets and biomarkers. However, the role of miRNA-mediated regulation of VMP1 in neurodegenerative diseases, hepatitis, pancreatitis, and viral infections remains understudied. The scarcity of reports in these areas suggests a significant gap in our understanding of VMP1’s broader physiological implications.

VMP1’s function as a host factor in coronavirus and flavivirus replication presents it as a potential broad-spectrum target for antiviral therapies. VMP1’s regulation of ER calcium may offer a novel strategy for controlling viral replication.

The discovery that VMP1 is subject to post-translational modifications, such as ubiquitination by the CRL4-DCAF2 complex, opens new avenues for therapeutic intervention.

In summary, VMP1 is a convergence point for various cellular processes, with profound implications for human health and disease. Its intricate regulatory network and diverse effects render VMP1 a promising target for future research and therapeutic development. Elucidating the complexities of VMP1’s functions and dysregulation will not only deepen our grasp of cellular biology but also facilitate the development of innovative clinical applications.

## 3 Conclusion

VMP1 emerges as a multifunctional protein with critical roles in cellular homeostasis, playing a significant part in autophagy, calcium regulation, and lipid metabolism. Its involvement in the pathogenesis of various diseases, including neurodegenerative disorders and cancer, underscores its potential as a therapeutic target. The intricate regulatory network in which VMP1 participates, along with its diverse cellular effects, positions it as a key node in the complex interplay of cellular processes. The modulation of VMP1’s activity, through its interaction with miRNAs and post-translational modifications, presents a nuanced approach to targeting this protein for therapeutic benefit.

### 3.1 Future directions

There is a clear need for a deeper understanding of VMP1’s molecular mechanisms and its role in disease pathology in order to develop targeted therapies. Future research should aim to.

1. Elucidate the physiological role, its regulation including post-translational regulation of VMP1 (ubiquitination, palmitoylation etc.) and its interaction with miRNAs, ER stress sensors could unveil new treatment strategies for a wider array of pathologies.
2. Identify VMP1’s roles and mechanisms in pathological conditions, such as inflammtion, tumor and neurodenerative disease, as well as viral infection.
3. Investigate VMP1’s potential as a biomarker for early diagnosis, prognosis, and treatment response across a range of pathologies.

By pursuing these research directions, the scientific community can unlock the full potential of VMP1 as a therapeutic target and enhance our knowledge of cellular biology, ultimately leading to innovative clinical applications that address a wide array of human diseases. Considering the close collaborative relationship between VMP1 and TMEM41B in physiological regulation, the potential redundancy between VMP1 and TMEM41B should be taken into account when considering VMP1 as a therapeutic target.
